# The interplay between orexin, neurodegeneration, cognition and sleep microarchitecture in mild to moderate Alzheimer's Disease

**DOI:** 10.1002/alz70856_107602

**Published:** 2026-01-09

**Authors:** Arsenio Paez, Gerard Piñol‐Ripoll, Shahla Bakian Dogaheh, Sam O Gillman, Farida Dakterzada, Anna Carnes, Ferran Barbe, Thien Thanh Dang‐Vu

**Affiliations:** ^1^ University of Oxford, Oxford, Oxfordshire, United Kingdom; ^2^ Centre de Recherche de l’Institut Universitaire de Gériatrie de Montréal, Montreal, QC, Canada; ^3^ Concordia University, Montreal, QC, Canada; ^4^ Institut de Recerca Biomédica de Lleida (IRBLLeida), Lleida, Spain; ^5^ Cognitive Disorder Unit, Hospital Universitari Santa Maria, Lleida, Spain; ^6^ Hospital Universitari Santa Maria de Lleida, IRBLleida, Lleida, Spain; ^7^ Hospital Universitari Santa Maria LLeida, LLeida, Spain; ^8^ Centre de recherche de l'Institut universitaire de gériatrie de Montréal, Montreal, QC, Canada

## Abstract

**Background:**

Cerebrospinal fluid (CSF) orexin levels are higher in MCI and AD and associated with sleep deterioration, increasing risk of cognitive decline and Alzheimer's disease (AD) progression. Orexin‐A is a key sleep‐wake cycle regulator. Dual orexin receptor antagonists improve sleep in AD and insomnia and may reduce tau and Aβ deposition in older adults. However, little research has investigated associations between sleep microarchitecture, orexin, neurodegeneration biomarkers, cognitive decline, or mental health in AD.

**Methods:**

Using data from a prospective cohort study of mild‐to‐moderate AD (*n* = 60, 30‐female, mean age‐74.7), we analysed non‐REM sleep spindles, slow oscillations (SO), and their associations with CSF orexin, AD biomarkers, cognition, and mental health over three years. Participants underwent polysomnography (PSG) and CSF draws at baseline, neuropsychological assessment with the Alzheimer's Disease Assessment Scale‐Cognitive Subscale (ADAS‐Cog) and Neuropsychiatric Inventory (NPI) at baseline and 12 months, and Mini‐Mental Status Examination (MMSE) at baseline, 12, 24, and 36 months.

PSG was scored along American Academy of Sleep Medicine guidelines. Spindle and SO detection were performed using in‐house, open‐source software packages developed at Concordia University, following Moelle (2011) recommendations for spindles and Staresina's (2015) recommendations for SO. Associations between SO and spindle characteristics (duration, density, power, amplitude) and orexin, Aβ42 and tau at baseline, and baseline orexin and cognition from baseline to 36 months were investigated with false discovery rate‐adjusted generalised linear models, controlling for age, sex, apnea‐hypopnea index.

**Results:**

We found previously unreported, predictive associations between SO, duration, density, amplitude, and CSF orexin. Orexin also predicted increased ptau181, total‐tau, ptau/Aβ42, total‐tau/Aβ42. Increased orexin predicted worse cognitive performance (higher ADAS‐cog, lower MMSE) from baseline to 36‐months and increased neuropsychiatric symptom severity (NPI) from baseline to 12 months. Orexin also moderates relationships between spindles, SO, cognition, and mental health.

**Conclusions:**

Orexin levels are associated with neurodegeneration biomarkers and cognitive deterioration in AD and moderate relationships between sleep microarchitecture and cognitive changes over time. Orexin may thus constitute a potential target for sleep‐related interventions for cognition in neurodegenerative disorders.

